# The quality of life in Alzheimer’s disease is not associated with handgrip strength but with activities of daily living–a composite study from 28 European countries

**DOI:** 10.1186/s12877-023-04233-1

**Published:** 2023-09-04

**Authors:** Rizwan Qaisar, M Azhar Hussain, Asima Karim, Firdos Ahmad, Fabio Franzese, Abeer A Al-Masri, Saad M Alsaad, Shaea Ayed Alkahtani

**Affiliations:** 1 Basic Medical Sciences, College of Medicine, University of Sharjah, Sharjah, United Arab Emirates; 2 Cardiovascular Research Group, Research Institute for Medical and Health Sciences, University of Sharjah, Sharjah, 27272 United Arab Emirates; 3 Department of Finance and Economics, College of Business Administration, University of Sharjah, Sharjah, 27272 UAE; 4 Department of Social Sciences and Business, Roskilde University, Roskilde, DK-4000 Denmark; 5 Exercise physiology department, college of Sport Sciences and Physical Activity, King Saud University, Riyadh, 11451 Saudi Arabia; 6 Department of Biomedical Sciences, College of Health Sciences, Abu Dhabi University, Abu Dhabi, 59911 United Arab Emirates; 7 SHARE Berlin Institute, Chausseestraße 111, 10115 Berlin, Germany; 8 Department of Physiology, College of Medicine, King Saud University, Riyadh, 11451 Saudi Arabia; 9 Department of Family and Community Medicine, College of Medicine, King Saud University, Riyadh, 11451 Saudi Arabia; 10 College of Sport Sciences and Physical Activity, King Saud University, PO Box: 2454, Riyadh, 11451 Saudi Arabia

**Keywords:** Alzheimer’s disease, Handgrip strength, CASP-12, SHARE

## Abstract

**Objectives:**

The relationship between handgrip strength (HGS) and quality of life is inconsistent. The purpose of this study was to investigate the potential association between HGS and quality of life in the settings of ageing and Alzheimer’s disease (AD).

**Methods:**

We investigated the HGS, CASP-12 (control, autonomy, self-realization, and pleasure) measure of quality of life, and physical capacity in European adults above 50, including controls (n = 38,628) and AD subjects (n = 460) using the survey of health, ageing, and retirement in Europe (SHARE; 2022).

**Results:**

AD subjects exhibited lower HGS and CASP-12 scores than controls (both p < 0.05). Participants with higher CASP-12 quartiles had higher HGS in controls but not in AD subjects. A linear positive relation was found between HGS and CASP-12 in controls (0.0842, p < 0.05) but not in AD subjects (0.0636, p = 0.091). There was no effect of gender on this finding. Lastly, we found significant negative associations of difficulties walking, rising from chair, climbing stairs, and fatigue with CASP-12 scores in controls and AD subjects (all p < 0.05).

**Conclusions:**

Altogether, HGS was not associated with quality of life in individuals with AD. Conversely, difficulties in activities of daily living seem to be negatively associated with quality of life; thus, strategies are recommended to improve physical capacity.

## Introduction

Quality of life (QoL) is a complex concept associated with the mental, social, and physical well-being of older adults [1]. Several studies indicate a high efficacy of QoL in predicting morbidity and mortality in older adults [2, 3]. For example, individuals with elevated QoL have reduced risks of hospitalization, comorbidities, and death [3]. These people also demonstrate improved functional independence in activities of daily living [3].

Several instruments are available to evaluate the QoL [1]. Among them, control, autonomy, self-realization, and pleasure (CASP-12) may be of primary relevance [4]. CASP-12 was designed for individuals over 50 years old with an intent to distinguish basic human needs. In this context, it differs from other QoL instruments that primarily focus on physical functions [5]. Over the years, CASP-12 has emerged as an efficient, objective, and standardized assessment tool to evaluate QoL in the older population [4]. In addition, it demonstrates adequate psychometric properties that go beyond the physical health of the individual [6]. The mental correlates of CASP-12 have been cross-culturally validated in several countries [4, 6]. However, the associations of CASP-12 with physical correlates are poorly characterized and warrant further dissection for targeted interventions.

Age-related muscle decline, termed sarcopenia, is a critical determinant of reduced QoL in the older population [7]. Sarcopenia is a geriatric syndrome characterized by the loss of muscle mass and strength and reduced physical capacity [8]. Reduced handgrip strength (HGS) is widely accepted among the diagnostic parameters of sarcopenia [9]. Several studies indicate an association of reduced HGS with compromised mobility, functional decline, hospitalization, and social isolation [10–12]. In addition, HGS exhibits positive associations with several QoL assessment tools [10]. However, most such studies are regional, and its association with CASP-12 in the settings of a large continental dataset is relatively poorly characterized.

Among various age-related comorbidities, Alzheimer’s disease (AD) is frequently associated with reduced QoL [6]. The subjects with AD also demonstrate reduced scores on CASP-12, indicating poor psychometric well-being [6]. The physical compromise in AD cohort is well established and also includes reduced HGS [13]. These findings indicate a potential association between CASP-12 and HGS in AD cohort. Specifically, it is possible that reduced psychometric well-being, as measured by CASP-12, may be associated with lower HGS. However, sarcopenia and muscle strength have multifactorial etiology, and the association of CASP-12 with HGS remains poorly characterized. Furthermore, such associations may be gender-specific, since gender differences exist in assessing QoL in older adults [14]. Lastly, most related studies investigate subsets of regional populations and may not represent large datasets. We aimed to investigate the associations between quality of life and HGS among more than 6000 European adults.

## Materials and methods

The Survey of Health, Ageing and Retirement in Europe (SHARE) is the dataset used for this study [15]. More specifically, wave 8 is used, which was conducted using face-to-face interviews from October 2019 until March 2020. SHARE is a representative harmonized panel-data survey covering 28 primarily European countries with the target population being people aged 50+. The SHARE collects data on different public health topics, socioeconomic factors, living conditions, as well as demographics, which enables drawing a complete picture of countries’ different vulnerable older adults.

The questionnaire behind our applied data is documented in the SHARE database [15]. In the accessed data sets applied here (datasets with suffix sharew8_rel8-0-0_: cv_r, gv_weights, ph, gv_health, and gv_isced), 46,487 respondents were identified. After cleaning the data for missing observations or missing values, we ended up with 38,628 individuals, which was a 17% reduction in sample size. The main problems behind missing values were with the quality of life and HGS indicators. Many interviews of respondents with AD were conducted with the help of a proxy, who assisted the respondent or answered some or all the questions on behalf of the respondent. Thus, it is possible that the respondents with AD in our dataset represent the healthier ones compared to the AD subjects in the whole data set. And we do see that among AD subjects in the reduced sample, much fewer suffer from walking and climbing difficulties compared to the whole sample. When estimating different parameters, data were weighted by applying household weights (variable cciw_w8_main), e.g., we used population weights giving us estimates for the adults above 50. It should be noted that SHARE data is de-identified (in terms of European law it is “pseudonymized”), such that respondents cannot be identified. In our study, we only report aggregated information, ensuring the anonymity of individual respondents.

HGS was assessed by the questionnaire’s grip strength (GS) module. The communication in this regard was: “Now I would like to assess the strength of your hand in a gripping exercise. I will ask you to squeeze this handle as hard as you can, just for a couple of seconds and then let go. I will demonstrate it now”; “I will take two alternate measurements from your right and your left hand. Would you be willing to have your handgrip strength measured?”; “Which is your dominant hand?”. The maximum value of all – up to four -measurements was used as an indicator of HGS if at least two valid measures for one hand were available and the two measures for the same hand did not differ more than 20 kg.

The subjective well-being of the respondents was based on 12 well-being indicators (three questions for each of the sub-scales control, autonomy, self-realization, and pleasure) introduced by phrasing, ‘’I will now read a list of statements that people have used to describe their lives or how they feel. We would like to know how often, if at all, you experienced the following feelings and thoughts: often, sometimes, rarely, or never”. The answers were given scores of 1 (often), 2, 3, and respectively 4 (never). The first indicator was “How often do you think your age prevents you from doing the things you would like to do?” and the last indicator was phrased as “How often do you feel that the future looks good for you?”. Thus, higher scores were associated with higher well-being. The composite index summarized in CASP-12 is the sum of the scores for each of the 12 indicators and thus varied frpm 4 (least possible well-being) to 48 (highest possible well-being).

In the questionnaire, after showing the respondent a list of 21 diseases/2 other options, AD was identified through the question “Has a doctor ever told you that you had/ Do you currently have any of the conditions on this card? [With this, we mean that a doctor has told you that you have this condition and that you are either currently being treated for or bothered by this condition”. The same method was used to identify hypertension, high cholesterol levels, diabetes, chronic lung disease, cancer, and chronic kidney disease.

One can expect differences between different socio-economic groups’ HGS-CASP-12-AD nexus. Therefore, statistical tests were carried out to investigate inter-group differences regarding well-being and hand grip strength. The standard t-test was used. For multivariate analysis a linear OLS regression was used to estimate the effects of AD and HGS on CASP-12.

## Results

The primary characteristics of the study population are summarized in Table 1. The sample consists of 16,935 males and 21,693 females. Apart from HGS, the distribution across different measures was mostly similar for males and females. A major part of the studied population was in their 60 or 70 s, while fewer were in their 50s or above 80. Around one-fourth had a tertiary education, while 1.2% of the population had AD. More than 40% had well-being levels between 40 and 48. 2.4% of males had HGS up to 20, while the corresponding percentage for females was 21.9%. Less than 2% of respondents had BMI of at least 40 and were labeled as severely overweight. Nearly half of the selected population suffered from hypertension, one-fourth had high plasma cholesterol levels, and 13–16% were diagnosed with diabetes mellitus. 8–10% of the study population had difficulty walking, 15–20% had difficulty getting up from a chair, and 9–13% had difficulty climbing one flight of stairs. 17–23% had fatigue problems. 6% suffered from lung disease, 5–7% had cancer, 14–24% had osteoarthritis, and 2% had kidney disease.

**Table 1:** Basic characteristics and the distribution of sample size in the study population (CASP-12; control, autonomy, self-realization, and pleasure, HGS; handgrip strength, BMI; body mass index, AD; Alzheimer’s disease)

Variable	Level	Males	Females	Males	Females
		%	%	n	n
Age (years)	50–59	11.7	14.9	1975	3223
	60–69	37.9	38.0	6418	8253
	70–79	34.6	32.1	5864	6955
	80+	15.8	15.0	2678	3262
Tertiary education	No	73.1	76.0	12,386	16,478
	Yes	26.9	24.0	4549	5215
Presence of AD	No	98.8	98.8	16,728	21,440
	Yes	1.2	1.2	207	253
CASP-12	Up to 19.99	0.2	0.4	39	77
	20-29.99	8.6	10.2	1456	2221
	30 = 39.99	45.6	47.0	7718	10,194
	40 or above	45.6	42.4	7722	9201
HGS (kg)	Up to 20	2.4	21.9	412	4754
	21–25	3.8	28.3	651	6141
	26–30	8.4	28.2	1425	6115
	31–35	14.7	15.4	2481	3333
	36 or above	70.7	6.2	11,966	1350
BMI (kg/m^2^)	Below 20	1.5	4.6	260	991
	20-29.99	76.1	71.1	12,890	15,429
	30-39.99	21.4	22.6	3616	4907
	40 or above	1.0	1.7	169	366
Hypertension	No	54.9	54.7	9302	11,872
	Yes	45.1	45.3	7633	9821
Cholesterol	No	75.0	74.4	12,698	16,132
	Yes	25.0	25.6	4237	5561
Diabetes	No	83.6	87.5	14,159	18,980
	Yes	16.4	12.5	2776	2713
Difficulty walking	No	92.1	90.0	15,589	19,523
	Yes	7.9	10.0	1346	2170
Difficulty getting up from chair	No	85.2	80.0	14,422	17,358
	Yes	14.8	20.0	2513	4335
Difficulty climbing one flight of stairs	No	90.6	86.8	15,351	18,832
	Yes	9.4	13.2	1584	2861
Fatigue	No	82.7	76.8	14,005	16,656
	Yes	17.3	23.2	2930	5037
Lung disease	No	93.6	94.2	15,851	20,424
	Yes	6.4	5.8	1084	1269
Cancer	No	94.3	95.3	15,964	20,675
	Yes	5.7	4.7	971	1018
Osteoarthritis	No	85.7	76.1	14,520	16,518
	Yes	14.3	23.9	2415	5175
Kidney disease	No	97.9	98.1	16,577	21,278
	Yes	2.1	1.9	358	415
Total		100	100	16,935	21,693

**Fig. 1:**
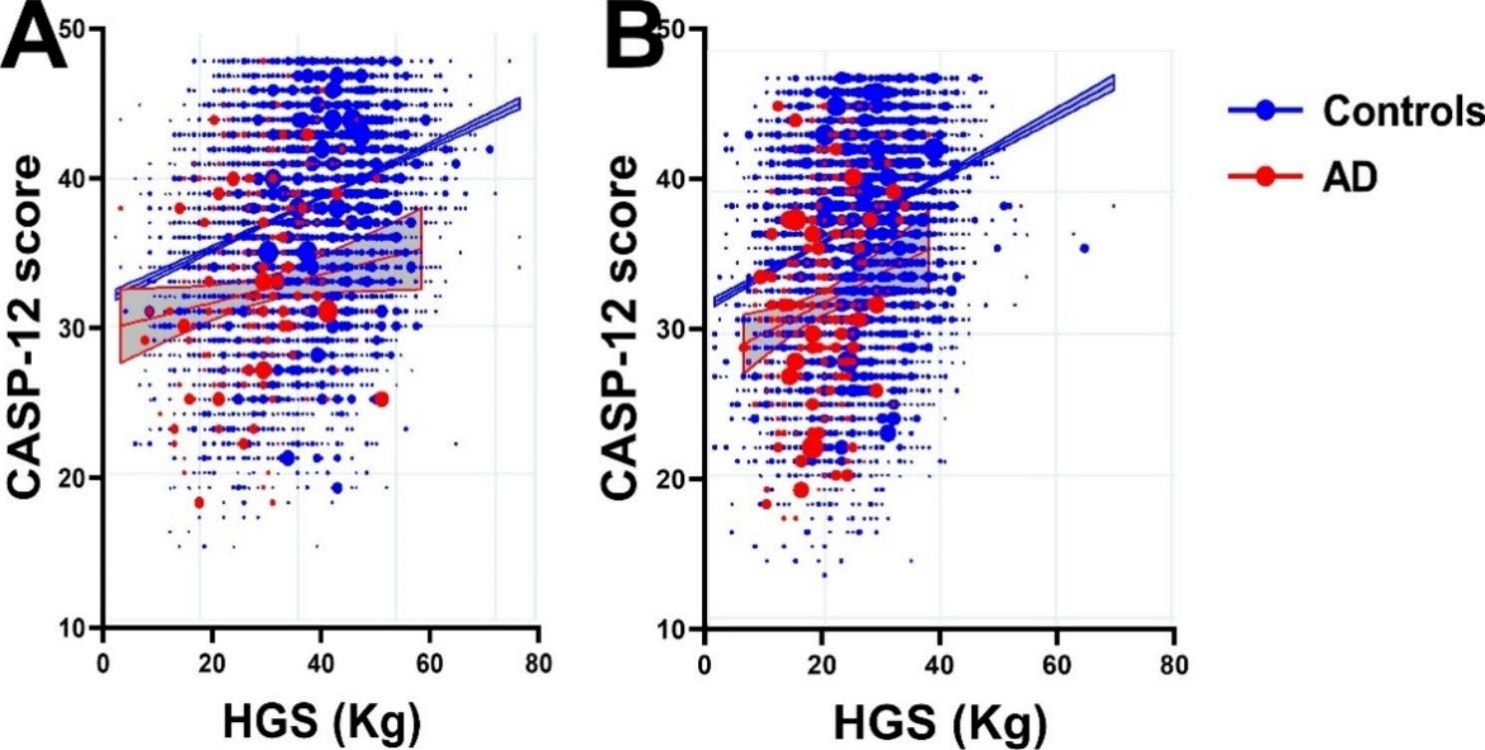
Linear regression analysis between CASP-12 scores and HGS in men (**A**) and women (**B**) controls and subjects with AD. (CASP-12; control, autonomy, self-realization, and pleasure, HGS; handgrip strength, AD; Alzheimer’s disease)

We next investigated the interplay of HGS and well-being with AD (Fig. 1). As expected, we observed that higher HGS was associated with higher scores on CASP-12 in males (Fig. 1A) and females (Fig. 1B). This relationship was more robust for non-Alzheimer’s respondents compared to AD subjects (the blue 95% confidence intervals were much narrower than the red CIs). Interestingly, the curves are steeper for females than males, meaning that a 1-point increase in HGS lead to a higher increase in CASP-12 for females than males.

Females had lower HGS than males, so we used different standardized thresholds for normal HGS for both genders, e.g., 27 kg for males and 16 kg for females. We observed that the well-being was mostly the same for males and females after applying HGS thresholds (Table 2). We also found systematic difference in CASP-12 scores between respondents above and below the thresholds, e.g., controls with normal HGS had CASP-12 around 38, while it was around 34 for respondents below normal HGS. But the difference was marginal among AD respondents. The difference between controls and AD was nearly 6 kg for normal HGS, which was highly significant (p < 0.001). There was also a difference between controls and AD for below-normal HGS, but it was 2 kg and failed to achieve statistical significance.

**Table 2:** Average CASP-12 scores by gender and HGS in controls and subjects with AD. *p < 0.05, **p < 0.01, ***p < 0.001. (CASP-12; control, autonomy, self-realization, and pleasure, HGS; handgrip strength, AD; Alzheimer’s disease)

		CASP-12				Sample size	
		Controls	AD	Difference		Controls	AD
Males	HGS ≤ 27	34.0	31.7	2.3		1389	65
	HGS > 27	38.7	32.8	5.9***		15,339	142
Females	HGS ≤ 16	33.8	31.6	2.2		1837	77
	HGS > 16	38.2	32.4	5.8***		19,603	176

Additionally, among control subjects, HGS was higher with higher CASP-12 quartile in males and females (Table 3). In the lowest CASP-12 quartile, HGS was 39.1 kg for males and 24 kg for females. Conversely, in the highest CASP-12 quartile, the HGS was 45.2 kg for males and 27.7 kg for females. However, a similar systematic relation between HGS and CASP-12 was not observed in AD respondents. For every CASP-12 quartile for both genders, we observed that the HGS was higher for controls than AD subjects, and the differences between the controls and AD subjects (5.3 to 11.4 kg) was highly significant (except for females in the CASP-12 third quartile, where the difference of 1.4 kg was not statistically significant).

**Table 3:** Average HGS by gender and CASP-12 quartiles in controls and subjects with AD. *p < 0.05, **p < 0.01, ***p < 0.001. (CASP-19; control, autonomy, self-realization, and pleasure, HGS; handgrip strength, AD; Alzheimer’s disease)

		HGS				Sample size	
		Controls	AD	Difference		Controls	AD
Males	CASP, Q1	39.1	32.7	6.4**		5183	122
	CASP, Q2	42.5	33.1	9.4**		3869	39
	CASP, Q3	44.6	34.6	10.0***		4259	34
	CASP, Q4	45.2	33.9	11.4**		3417	12
Females	CASP, Q1	24.0	18.7	5.3***		6359	159
	CASP, Q2	26.0	19.8	6.3***		5927	47
	CASP, Q3	27.2	25.8	1.4		3959	22
	CASP, Q4	27.7	19.8	7.8***		5195	25

In the above descriptive analysis, we did not control for comorbidities or demographics, such as age, educational level, and the country of residence. All of this is considered in the CASP-12 regressions presented in Table 4. In the overall regression, we observed that higher HGS was associated with higher CASP-12 scores. For example, a 1-point increase in HGS was associated with a 0.0821 point higher CASP-12. The presence of AD was associated with lower CASP-12 by 1.569. Additionally, AD reduced the relation between HGS and CASP-12 by 0.0452. For example, the sensitivity of CASP-12 to HGS was roughly halved from 0.0821 to 0.0369, albeit it was not statistically significant. After applying regression between controls and AD subjects separately, we observed that the relationship between HGS and CASP-12 was nearly unchanged for controls (1-point increase in HGS lead to a 0.0809 increase in CASP-12) and AD subjects (1-point increase in HGS lead to 0.0793 increase in CASP-12). In separate regressions for males and females, we found robust relationship between HGS and CASP-12 for controls (0.0773 for males and 0.1010 for females) and a relatively modest and statistically insignificant relation for AD (0.0735 for males and 0.0722 for females). Together, these observations show a lack of association between HGS and well-being for AD subjects.

**Table 4:** Regression values of CASP-12 with HGS, gender, age, and various motor disabilities in controls and subjects with AD. *p < 0.05, **p < 0.01, ***p < 0.001. (CASP-12; control, autonomy, self-realization, and pleasure, HGS; handgrip strength, AD; Alzheimer’s disease)

	Males and females			Males		Females	
	Alle	Without Alzheimer’s	With Alzheimer’s	Without Alzheimer’s	With Alzheimer’s	Without Alzheimer’s	With Alzheimer’s
HGS	0.0821***	0.0809***	0.0793*	0.0773***	0.0735	0.101***	0.0722
Alzheimer’s	-1.569*	.	.	.	.	.	.
Alzheimer’s and HGS	-0.0452	.	.	.	.	.	.
Female	1.051***	1.027***	1.844**	.	.	.	.
Age	0.202***	0.233***	-1.668***	0.314***	-1.216	0.212***	-2.107***
Age squared	-0.157***	-0.179***	1.124***	-0.223***	0.794	-0.173***	1.424***
Walking difficulty	-1.976***	-2.010***	-0.766	-2.044***	-1.451	-1.925***	-0.690
Getting up from chair diff.	-1.454***	-1.456***	-1.543**	-1.644***	-1.385	-1.296***	-2.267*
Climbing one flight diff.	-1.796***	-1.784***	-2.125***	-1.676***	-0.270	-1.771***	-2.996***
Fatigue	-3.189***	-3.195***	-2.914***	-3.421***	-2.886**	-3.053***	-2.846***
Constant	32.21***	31.21***	96.15***	27.73***	82.58**	32.90***	113.5***
N	38,628	38,168	460	16,728	207	21,440	253
R-sq	0.271	0.265	0.355	0.252	0.338	0.277	0.468

Note: Transformed weights are used such that there is representativeness within countries, while at the same time all countries weigh equally

## Discussion

We report that the reduced HGS and CASP-12 were common findings in subjects with AD. These subjects also exhibited difficulties performing several household motor tasks compared to controls. We found significant correlations between difficulties in performing household tasks and CASP-12 scores in AD subjects. However, to our surprise, we did not find a similar robust correlation of HGS with CASP-12 in AD subjects. This finding was consistent across both genders and indicates that HGS may not be primarily associated with CASP-12 in AD.

The reduced HGS and difficulties in performing daily motor tasks in AD subjects is consistent with previous reports [13, 16]. This is partly due to accelerated neurodegeneration, which affects skeletal muscle and motor systems. In addition, these subjects also have reduced physical activity, which may further exacerbate neural and/or motor decline. Accordingly, several reports indicate an association between sarcopenia and CASP-12 scores in older adults [7, 17]. Thus, we expected a robust negative association between HGS and CASP-12 scores in AD subjects. However, our finding of a weak association between HGS and CASP-12 in AD subjects is counterintuitive. It must be noted that HGS is a measure of upper body strength and may have a relatively lesser role in ensuring functional independence and quality of life than lower body strength. For example, several activities of daily living, such as walking, rising from the chair, and climbing stairs, primarily depend on lower limb strength with minimal contribution from handgrip muscles [18]. It is generally recognized that the activities requiring lower extremities strength are lost earlier than the upper limb strength in individuals with AD [19, 20]. Thus, the discriminatory role of HGS in mobility and lower body strength may not be well established and is reduced in subjects with optimal muscle strength [21]. A functionally independent lifestyle is a critical driver of higher CASP-12 scores in older population [4]. For example, subjects with difficulty in performing activities of daily living exhibit reduced CASP-12 scores [7]. Thus, we next investigated the associations of CASP-12 with difficulties in performing activities of daily living. We found consistent and robust negative associations of CASP-12 with difficulties in walking, getting up from chair, and climbing stairs and a higher fatigue sensitivity. These novel findings in AD subjects are generally consistent with the efficacy of CASP-12 in predicting physical dependency in older adults [7].

Previous studies also indicate the associations of CASP-12 with sarcopenia in older population [7, 17]. We did not evaluate sarcopenia in the study population. However, we measured HGS, which exhibited a weak association with CASP-12 in AD subjects. It must be noted that sarcopenia is a composite syndrome with multiple causative factors and phenotypes [22–24]. A low grip strength is an individual component of sarcopenia [25]. In addition, the HGS measurements do not consider muscle power, which may be a stronger predictor of physical capacity. Findings from the older population indicate a disproportionate loss of muscle strength between upper and lower extremities [18]. On the other hand, the loss of muscle power is generally consistent between upper and lower limbs [18]. Lastly, it must be noted that CASP-12 intends to distinguish basic human needs, unlike other QoL tools, which rely on physical health. Thus, the reduced CASP-12 scores in AD subjects may primarily be independent of reduced HGS. Together, these findings may translate into the absence of a robust association between HGS and CASP-12 in AD subjects.

Contrary to AD subjects, the control population exhibited robust associations of CASP-12 with HGS. However, the control population also exhibited higher HGS than AD subjects. It is possible that at higher HGS, the CASP-12 is at least partly governed by HGS. This notion is consistent with the positive association of HGS with mental health in older adults [26]. Additionally, HGS also demonstrates a positive association with physical health in advanced age [26]. However, these effects may partly be dependent on the amount of HGS. For example, at lower HGS, the lower limb strength and difficulties in performing daily motor tasks become the prime determinants of CASP-12 [21]. Most previous studies dissecting the association between HGS, and quality of life overlook the lower limb functioning. In general, older adults with lower limb dysfunction also exhibit lower HGS. However, some level of discord between the functions of lower and upper limb is reported [18]. It is possible that this discord can at least partly explain the reduced predictive potential of HGS in describing CASP-12 in AD subjects.

We found relatively lower CASP-12 scores among women than men irrespective of disease status, which is consistent with recent data from Europe [27]. Women with low HGS are more likely to have reduced psychosocial health than men [28]. In addition, women exhibit a more rapid decline of lower extremities strength and mobility than men [21]. Together, these findings may account for lower CASP-12 scores in women than men.

The data presented in this study represent a 17% reduction from the original dataset of 46,487 subjects. This was primarily due to missing data about HGS and CASP-12. This reduction did not significantly change the age and gender distribution of subjects but reduced the prevalence of AD from 2.02 to 0.82%. AD subjects exhibit reduced mental and physical well-being compared to age-matched controls [6, 13, 16]. For example, they demonstrate depression, reduced cognition, and muscle weakness for the given age. Thus, it is possible that the physical and mental decline in AD subjects may have prevented obtaining reliable data regarding HGS and CASP-12.

Pain is a critical determinant of QoL. Older adults may suffer from neuropathic pain due to comorbidities and somatosensory disturbances [29]. It is generally recognized that pain affects several QoL domains, including physical and emotional health [30]. It is possible that the potential presence of pain sensation may have affected the QoL data reported in this study. However, various domains of CASP-12 do not evaluate pain sensation. Therefore, we did not investigate pain sensation as a potential confounding factor in this study.

The major strengths of this study are a large sample size of participants with representative proportions population from various geographical regions of Europe. The diversity of the study population increases the global relevance of our findings. The HGS dynamometer is an objective tool and can be used in most clinical and domestic settings without technical expertise. We applied the CASP-12 as an internationally recognized measure of psychometric health standardized across the study population. However, this study has certain limitations. Any causal inference between physical and mental health should be cautiously interpreted due to the cross-sectional design of this study. We cannot validate the temporal stability of our findings due to lack of longitudinal data. We overlooked the comorbidities of participants in the control group. The data from some AD subjects was collected with the help of a proxy, which can potentially bias our results. However, SHARE is an internally recognized platform, and we are confident that all data was collected and validated in a standardized manner. Therefore, we are confident about the quality of our data. Lastly, it is possible that a significant number of AD subjects were not included in this study due to lack of data regarding HGS and CASP-12. Thus, the final sample size in this study may represent a selected pool of AD subjects from European countries.

Altogether, we report a reduction in HGS and CASP-12 in European adults with AD. However, HGS was not primarily associated with reduced CASP-12 in AD subjects. Conversely, the difficulties in performing daily motor tasks emerged as superior tools for dictating mental health in these subjects. Future studies should further characterize the physical and mental health in AD with mechanistic perspectives.

## Data Availability

SHARE Data is available free of charge for scientific purpose. Data access can be applied by the SHARE Research Data Center, see SHARE website: https://share-eric.eu/data/data-access. Access to the data collected and generated in the SHARE projects is provided free of charge for scientific use globally, subject to European Union and national data protection laws as well as the publicly available Conditions of Use. As long as the data is available for everyone, there is not necessary to contact and chase permission from authors. However, should researchers need further information or understanding the process, Dr Fabio Franzese (one of the authors of the current study) can be contacted.
